# ASO Author Reflections: ICG Fluorescent-Guided Surgery for Sentinel Lymph Node Biopsy Used as a Single Tracer Shown Sensitivity, Safety, and Efficacy in Early Breast Cancer Surgery

**DOI:** 10.1245/s10434-024-16266-w

**Published:** 2024-09-23

**Authors:** Vassilis Pitsinis, Rahul Kanitkar, Alessio Vinci, Wen Ling Choong, John Benson

**Affiliations:** 1 Department of Breast Surgery, NHS Tayside, Dundee, UK; 2 Faculty of Medicine, University of Dundee, Dundee, UK; 3 Breast Unit, Cambridge University Hospitals NHS Foundation Trust, Cambridge, UK; 4 School of Medicine, University of Cambridge, Cambridge, UK; 5 Anglia Ruskin University, Cambridge, UK; 6 Edinburgh, UK

## Past

The advent of sentinel lymph node biopsy has revolutionized surgical staging of the axilla in patients with early stage breast cancer. Targeted sampling relies on identification of sentinel lymph nodes (SLN) using some form of tracer agent taken up by the first draining nodes. Initial studies of the SLN biopsy technique employed blue dye, radioisotope, or a combination thereof. There are potential drawbacks from both conventional tracers including allergic reactions, staining of tissues, cumulative radiation exposure, problems with surgical waste disposal, and mandatory licensing. Moreover, radioisotopes are a biproduct of a contracting nuclear industry and supply might become unpredictable with greater usage globally, particularly within emerging Asian economies. Localization techniques using alternative tracers warrant investigation and fluorescent imaging of lymphatic tissue with indocyanine green (ICG) combined with either blue dye^1,2^ or radioisotope^3^ has equivalent performance indicators but improved safety profile with infrequent allergic reactions.

## Present

This strategy of combining ICG with either blue dye or radioisotope as a co-tracer represents a transition phase permitting accrual of clinical experience with fluorescence while striving toward reliance on ICG as a sole tracer agent, thereby avoiding disadvantages of conventional tracers that also include cost and availability. Tracer combinations with dual localization are more expensive and inconvenient than ICG alone and not necessarily associated with improved accuracy that is meaningful in terms of key clinical outcomes.^4^ A limited number of observational studies have evaluated ICG as a single tracer for SLN biopsy in patients with breast cancer and shown high levels of sensitivity and efficacy with similar node positivity rates and no serious adverse effects. This paper reports results of the first randomized trial comparing a dual combination of ICG and a conventional tracer with ICG alone.^1^ Although a relatively small trial, ICG alone was found to be non-inferior to combination with either blue dye or radioisotope and provides additional support for the efficacy and safety of ICG alone for routine SLN biopsy in patients with early breast cancer.

## Future

It is hoped that ICG will be more readily utilized by the breast surgical community with raised awareness of its advantages. The competitive benefits for ICG usage include being cost effective (although upfront cost for a fluorescent camera can be considerable), short learning curve, very low allergic reaction risk, shortage of radioisotope, and higher blue dye allergic reaction and tissue staining, as well as flexibility of the technique (can be used across medical and surgical specialties, e.g., to check flap perfusion). Proposals for future work include (1) larger follow-up randomized trial to substantiate findings from the current study on ICG as a sole tracer; (2) using ICG as a single tracer in the neoadjuvant chemotherapy setting; and (3) increased competition among producers of fluorescent imaging systems will drive equipment prices down, allowing for widespread adoption by surgical communities, including low- and middle-income countries.
